# Supplementary figures and images for: A nonsense mutation in the PRKG2 gene in dalmatian dogs with chondrodysplasia
Source: PLoS One. 2025 Nov 26;20(11):e0322107. doi: 10.1371/journal.pone.0322107 (PMC12654895; doi:10.1371/journal.pone.0322107)

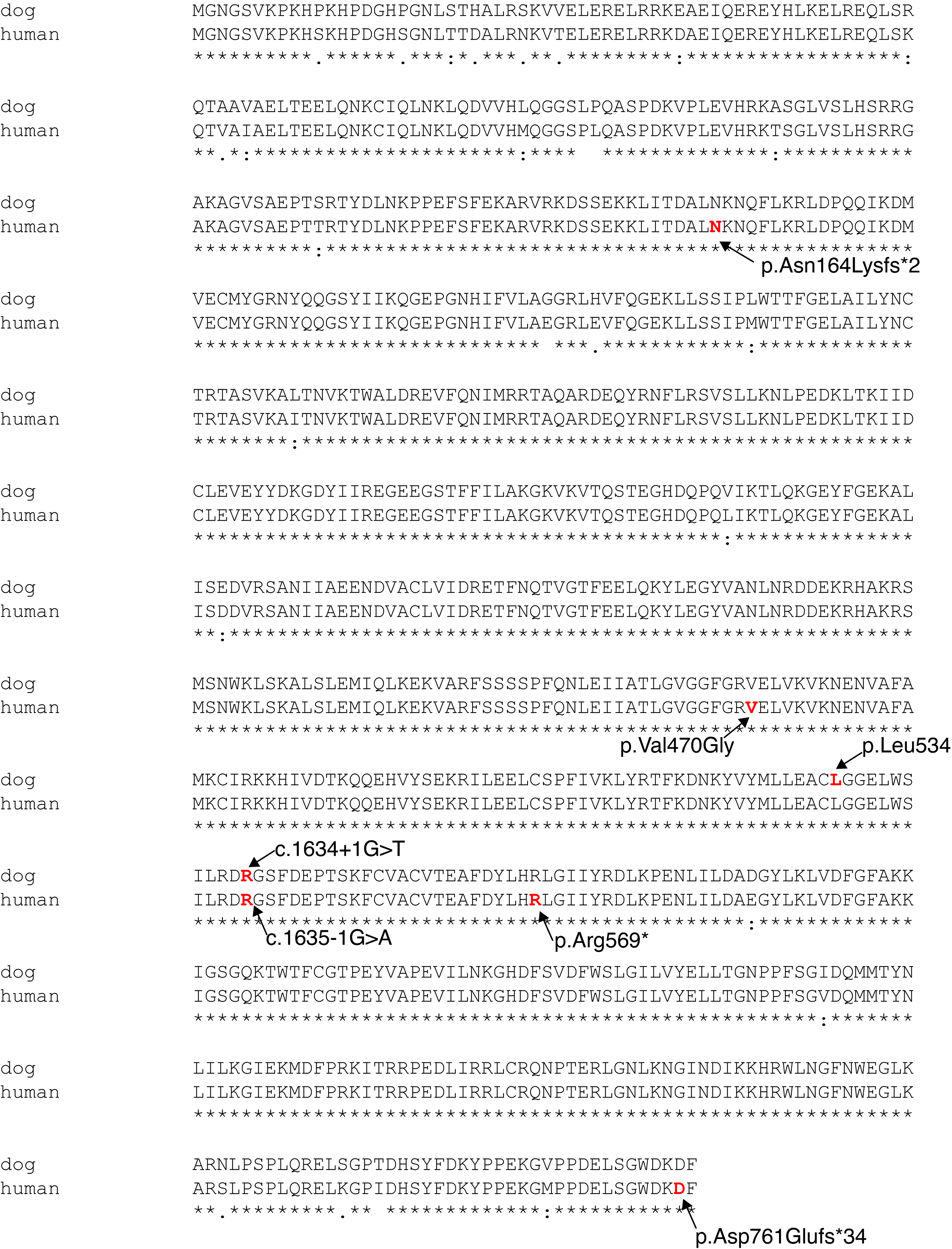

Supplement: S1 Fig — The pairwise alignment (Needle) of the human (UniProtKB accession number Q13237-1) and canine (UniProtKB A0A8C0NE14) 762 amino acid long PRKG2 protein show 96.7% (735/762) identity. The identified nonsense variant at position 534 in this study as well as the previously described splice donor variant in Dogo Argentino is indicated on the canine sequence as well as disease-causing variants in human patients. (TIF) [file pone.0322107.s002.tif]

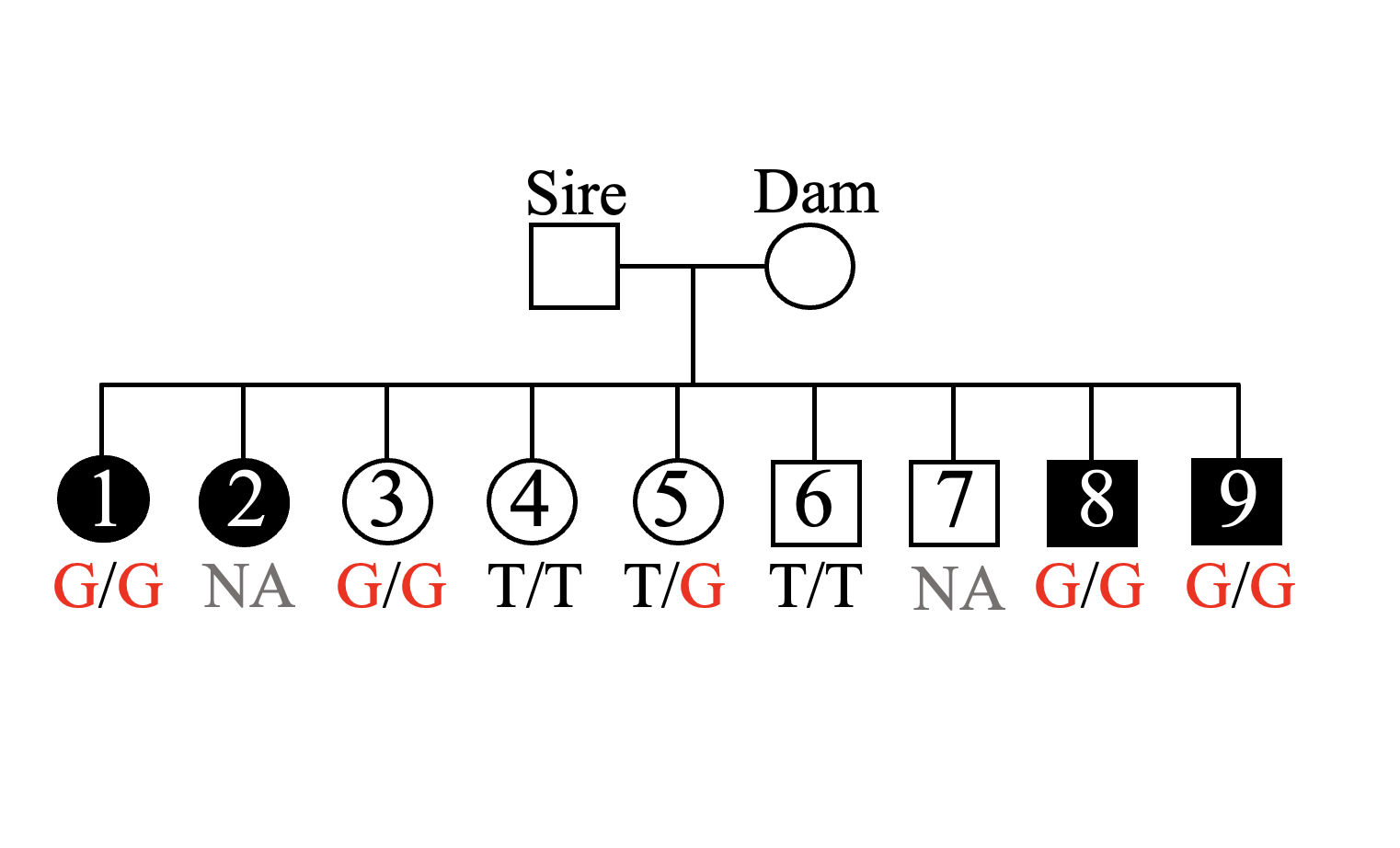

Supplement: S2 Fig — Pedigree showing the unaffected parents and nine offspring. Offspring 1 was clinically affected and included in the clinical study. Offspring 2 was clinically affected and included in the clinical study, but no blood sample was available for this dog. Offspring 3 was not clinically examined and assumed healthy. According to the owner this dog was small and sturdier than normal. Offspring 4 was healthy and clinically examined at the age of four months. Offspring 5 and 6 were assumed healthy and were not clinically examined. Offspring 7 was assumed healthy and no blood sample was available for this dog. Offspring 8 and 9 were clinically affected and whole-genome sequenced. Offspring 9 was included in the clinical study. Circles represent females, squares represent males. G/G = homozygous for the PRKG2 nonsense mutation. T/G = heterozygous for the PRKG2 nonsense mutation. T/T homozygous wildtype. (TIF) [file pone.0322107.s003.tif]
